# Evaluation of the efficacy of radiofrequency ablation therapy in cases with parathyroid adenoma

**DOI:** 10.55730/1300-0144.6108

**Published:** 2025-10-10

**Authors:** Bahri EVREN, Abdulkadir BOZBAY, İsmail Okan YILDIRIM, Ömercan TOPALOĞLU, İbrahim ŞAHİN

**Affiliations:** 1 Department of Endocrinology, Faculty of Medicine, İnönü University, Malatya, Turkiye; 2 Department of Endocrinology, Yozgat City Hospital, Yozgat, Turkiye; 3 Department of Radiology, Faculty of Medicine, İnönü University, Malatya, Turkiye; 4 Department of Internal Medicine, Faculty of Medicine, Bülent Ecevit University, Zonguldak, Turkiye; 5 Department of Endocrinology, Faculty of Medicine, Memorial Şişli Hospital, İstanbul, Turkiye

**Keywords:** Primary hyperparathyroidism, parathyroid adenoma, radiofrequency ablation, hypercalcemia

## Abstract

**Background/aim:**

Radiofrequency ablation of solitary parathyroid adenoma has been used to treat primary hyperparathyroidism (pHPT) in high-risk patients for parathyroidectomy. This study aimed to evaluate the clinical efficacy of radiofrequency ablation for treating pHPT in patients with parathyroid adenomas.

**Materials and methods:**

The sample for this retrospective study comprised all consecutive patients with solitary parathyroid adenoma treated with radiofrequency ablation between 2013 and 2021. Patients’ baseline serum calcium and parathyroid hormone (PTH) values were obtained. The patients were followed up with serial biochemical measurements after the intervention, then at the first-week, first-month, third-month, sixth-month, and twelfth-month follow-ups. The study’s primary outcome was a biochemical cure, defined as the reestablishment of normal serum calcium and PTH levels, persisting for at least 6 months after the ablation.

**Results:**

The changes in the serum calcium and PTH levels were significant (p < 0.05). The biochemical cure rate was 30.2% at 1 year. At the end of 1 year, the rate of patients with normal serum calcium levels was 86.6%, whereas the rate of patients with normal serum calcium and higher PTH levels was 55.8%.

**Conclusion:**

Radiofrequency ablation of a solitary parathyroid adenoma may be considered an alternative treatment for pHPT, given that more than half of the cases investigated in this study had normocalcemic hyperparathyroidism at 1 year.

## 1. Introduction

Primary hyperparathyroidism (pHPT) secondary to solitary or multiple parathyroid adenomas is characterized by the persistent elevation of parathyroid hormone (PTH) and serum calcium levels [1]. Although most patients with pHPT are asymptomatic, a variety of symptoms such as pathological bone loss, nephrolithiasis, neurocognitive impairment, and hypercalcemic crisis may accompany pHPT in symptomatic cases [1–3]. Surgical removal of diseased parathyroid glands has been regarded as the preferred standard treatment modality for all symptomatic and selected asymptomatic pHPT patients [1]. Nevertheless, parathyroidectomy is not devoid of complications such as recurrent laryngeal nerve injury, hypoparathyroidism with hypocalcemia, and postoperative scar [1,4]. Ineligibility of senile patients for surgical treatment or refusal of treatment is another motive for developing minimally invasive, nonsurgical percutaneous modalities [2,5,6].

For the last several decades, ultrasound-guided percutaneous thermal ablation methods, including radiofrequency ablation (RFA), microwave ablation (MWA), high-intensity focused ultrasound, and laser, have been used as alternative treatment modalities for pHPT [1,2,7–11]. Ultrasound-guided ethanol injection has also been used [10,12,13]. Decreased complication rates and reduced need for anesthesia are the desired outcomes of alternative treatment modalities [1]. RFA has been used to ablate benign thyroid nodules and primary and metastatic tumors of the liver, lung, bone, and kidney, with acceptable safety and efficacy [1,5]. Good short- and long-term outcomes have been achieved with RFA in the treatment of pHPT, yet in some cases, clinical complications, such as abnormal PTH and serum calcium levels, have developed [1].

Few studies in the literature have focused on RFA for treating parathyroid adenomas causing pHPT [1,2,4,10,14,15,16]. In addition, the sample sizes and follow-up periods of these studies did not allow for definitive evidence [8,10,17]. In this context, the objective of this study is to evaluate the clinical outcomes of RFA for pHPT using a larger sample size and a longer follow-up period.

## 2. Materials and methods

### 2.1. Research design

This study was a retrospective analysis of all patients with solitary parathyroid adenoma who underwent RFA for pHPT between 2013 and 2021 at the İnönü University Faculty of Medicine, Department of Radiology, in Malatya. The local ethics committee approved the study protocol on 19 February 2019 (decision number 26). Written informed consent could not be obtained from the patients due to the study’s retrospective design and the anonymity of the data. The study was carried out in accordance with the principles outlined in the Helsinki Declaration.

### 2.2 Population and sample

The study population consisted of patients diagnosed with pHPT according to the criteria set forth at the Fourth International Workshop on Primary Hyperparathyroidism [18]. Imaging of the parathyroid glands was performed using ultrasound and technetium 99m sestamibi (Tc-99m methoxy isobutyl isonitrile). All symptomatic and asymptomatic patients with one of the following criteria were included in the study: a) hypercalcemia; b) T-score of – 2.5 or less at the lumbar, spine, femoral neck, total hip regions, or distal one-third of the radius bone, or increased risk of fragility; c) creatinine clearance of < 60 mL/min and/or nephrocalcinosis confirmed via imaging; d) age of < 50 years. As an institutional policy, surgical treatment of pHPT with parathyroidectomy was the standard treatment modality. The ultrasound-guided percutaneous RFA was offered to patients who were not eligible for, or refused, surgical treatment for pHPT. A council of surgeons and endocrinologists evaluated the surgical eligibility of these patients and determined they were not eligible for surgery. The exclusion criteria were recurrent or secondary hyperparathyroidism, history of neck surgery, clinically suspected malignant tumors, and coagulation disorders. Patients who were followed up for less than 12 months were excluded from the study. In the end, the study sample comprised 43 patients with pHPT treated with RFA. The mean age of the study sample, comprising 31 (72.1%) females and 12 (27.9%) males, was 57.7 ± 15.9 years.

### 2.3. Interventions

All procedures on parathyroid imaging and ultrasound-guided tumor ablation were performed by a single experienced physician using ultrasonography with a GE ML6-15-D probe. The technical details of the procedure were previously described in the literature [1,2,4].

### 2.4. Variables and follow-up

The PTH and serum calcium levels were measured 1 week before the procedure. The laboratory tests were repeated after the intervention and then at the first-week, first-month, third-month, sixth-month, and twelfth-month follow-ups. The study’s primary outcome was a biochemical cure, defined as the reestablishment of normal serum calcium and PTH levels, persisting for at least 6 months after the ablation [1,2,14]. Accordingly, treatment failure was defined as the absence of normal PTH and serum calcium levels for 6 months or more [10].

### 2.5. Statistical analysis

Descriptive statistics were expressed as mean ± standard deviation values in the case of continuous data that were determined to conform to the normal distribution, as median and minimum–maximum values in the case of continuous data that were determined not to conform to the normal distribution, and as numbers and percentages in the case of categorical variables.

The Friedman test was used to analyze the changes in serum calcium and PTH levels over time. The Durbin–Conover test was used to determine the intervals that yielded significance in multigroup comparisons.

Jamovi (version 2.3.12, 2022; https://www.jamovi.org) and Jeffreys’ Amazing Statistics Program (JASP, version 0.16.2, 2022; https://jasp-stats.org) were used for the statistical analysis. Probability (p) values of ≤0.5 were deemed to indicate statistical significance.

## 3. Results

**Figure 1:**
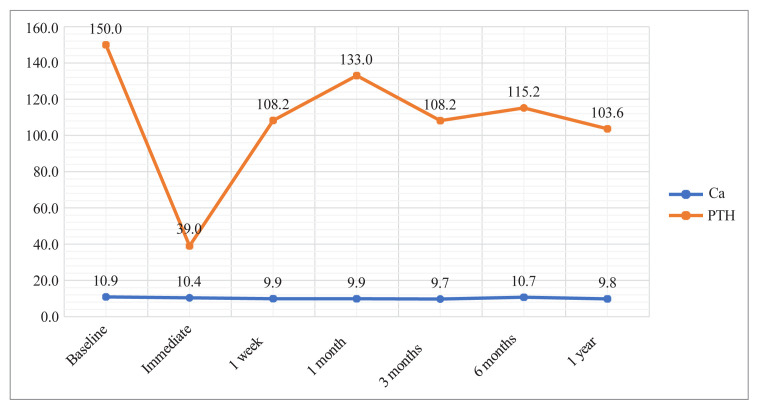
Changes in serum calcium and parathyroid hormone levels over time during the follow-up time of 1 year.

The mean preoperative calcium and PTH values for patients were 10.9 ± 0.7 mg/dL and 208.4 ± 163.0 pg/mL, respectively (Table 1). The changes in calcium and PTH levels over the study period are shown in Figure 1. Significant decreases in serum calcium levels were observed after the intervention. Accordingly, the mean serum calcium level decreased from 10.9 mg/dL to 9.7 mg/dL at the postinterventional third-month follow-up and remained stable until the 1-year follow-up. The median serum calcium levels at each postinterventional follow-up were significantly lower than the preinterventional median (p < 0.001 for all).

The median PTH level decreased significantly from 150.0 pg/mL to 39.0 pg/mL immediately after the intervention; however, it increased back to 133.0 pg/mL by the first-month follow-up and then remained steady over the study period. Yet, the median PTH levels determined in each of the postinterventional follow-ups were significantly lower than the preinterventional median PTH level (p < 0.05 for all) (Table 1).

At the end of the 1-year follow-up period, 13 (30.2%) patients achieved biochemical cure, characterized by normal serum calcium and PTH levels, whereas treatment failed in 30 (69.8%) patients.

**Figure 2:**
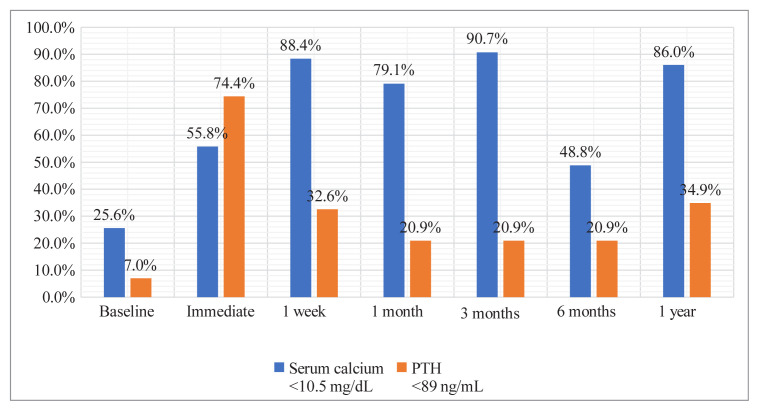
Frequencies of patients with post-interventional normal serum calcium and parathyroid hormone levels.

**Figure 3:**
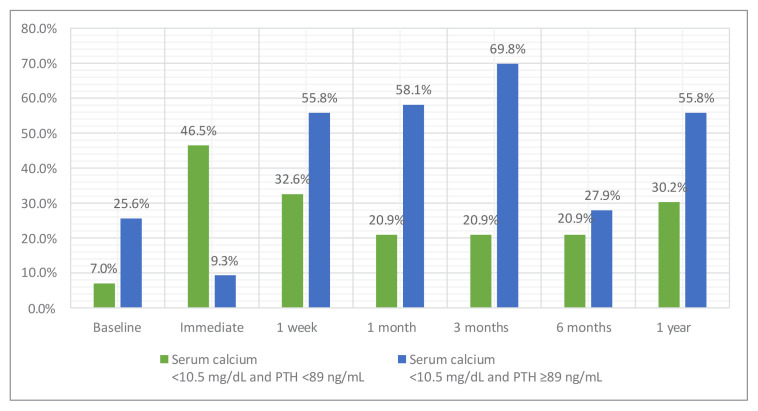
The proportion of the patients with normal serum calcium and parathormone levels (Green histogram) and normocalcemic hyperparathyroidism (Blue histogram).

The frequencies of the patients with normal postinterventional serum calcium and PTH levels are given in Table 2 (Figure 2). The highest rate of patients with normal calcium levels was detected in the third-month follow-up. An abrupt decrease was observed in the rate of patients with normal PTH levels from 74.4% after the intervention to 32.6% in the first week of follow-up. On the other hand, the proportion of patients with normal serum calcium levels gradually increased from 55.8% after the intervention to 86.6% at 1-year follow-up (Figure 2). The rate of patients with normal serum calcium and high PTH levels at the end of 1 year was 55.8% (Table 2, Figure 3).

## 4. Discussion

The use of RFA for the treatment of solitary parathyroid adenoma resulted in normal serum calcium levels in most patients within 1 year of the intervention. Nevertheless, the PTH levels of these patients increased gradually during the same period. Consequently, more than half of the patients had normocalcemic hyperparathyroidism one year after RFA.

There are many studies in the literature that compared the use of various ablative techniques in the treatment of pHPT [1]. MWA has been the most widely used technique in these studies [15,19,20]. To give a few examples, Liu et al. [21] compared MWA with surgical parathyroidectomy in the treatment of pHPT and found no significant difference between the success rates of the two approaches, although the cure rate was higher in the parathyroidectomy group (89.3%) than in patients with MWA (82.1%). Wei et al. [2] also did not find any significant difference in safety or effectiveness between MWA and RFA. There is no study in the literature that compared RFA with parathyroidectomy in the treatment of pHPT. Prospective studies evaluating the efficacy of various ablative methods and parathyroidectomy are needed to identify the most effective treatment for pHPT.

Different criteria have been used in the literature to define outcomes of ablative procedures for pHPT, including procedural/operative success rates, complete clinical cure, and technical success [1]. Most authors defined biochemical cure after RFA as normal postinterventional serum calcium and PTH levels that persist for at least 6 months [1,2,10,19,22]. In a study of 25 patients, Li et al. [1] reported a biochemical cure rate of 84% at 6 months after RFA. On the other hand, the biochemical or clinical cure rates reported at 6-month or 1-year follow-ups ranged from 63.6% to 98% across studies [1,2,4,10,14,16]. Other studies with relatively few patients or case reports have reported similar outcomes [4,9,10,13,15,17,22–25]. In contrast, the biochemical cure rate was found as 30.2% 1 year after the intervention in this study.

Longer follow-up times have been associated with reduced clinical cure rates following ablative interventions. For example, in a study featuring MWA treatment, the rates of patients with normal serum calcium and PTH levels after MWA were 80.0% and 62.5% at the 12- and 24-month follow-up visits, respectively [6].

Other studies have reported an abrupt reduction in serum calcium and PTH levels following RFA [1,14,16]. Similarly, the rates of patients with normal serum calcium and PTH levels after the intervention in this study were found as 55.8% and 74.4%, respectively. However, the biochemical test results measured in this study changed in the opposite direction over time. Accordingly, the serum calcium levels continued to decrease slowly from the first-week follow-up to the 1-year follow-up; however, PTH levels did not accompany this decrease. Zhang et al. [20] suggested that incomplete destruction of the hyperfunctioning parathyroid gland may be the primary reason for slightly higher PTH levels. They also speculated that MWA caused the gradual destruction of parathyroid tissue, leading to ongoing limited PTH secretion. The stabilization of PTH levels during the follow-up period supported this hypothesis. The capacity of parathyroid cells to proliferate may be another factor contributing to the high PTH levels [11]. In fact, Li et al. [1] found that patients without a cure also had normal serum calcium levels despite elevated PTH levels.

Recent studies focused on the rate of normocalcemic hyperparathyroidism following RFA. In parallel, two studies conducted in China [14,16] reported the rates of patients with normocalcemic hyperparathyroidism at 3 months and 1 year after RFA as 30% and 44.7%, and 12.5% and 16%, respectively. In comparison, the rate of patients with normocalcemic hyperparathyroidism was found to be 55.8% 1 year after the intervention in this study. As in parathyroidectomized patients, eucalcemic PTH elevations have also been detected in patients with ablated parathyroid adenoma. In addition, it has been speculated that multigland disease, parathyroid hyperplasia, or larger adenoma size are independent risk factors for treatment failure associated with ablative procedures [1]. In this context, parathyroid gland sizes of 0.6–0.7 cm or greater were considered an independent risk factor for uncured pHPT [1–3]. Parathyroid gland size was not assessed in this study; however, the presence of multiglandular disease detectable by imaging was used as an exclusion criterion. Therefore, inadequate destruction of the parathyroid adenoma cells might, in fact, be the primary reason for the relatively low biochemical cure and higher normocalcemic hyperparathyroidism rates in this study.

The sample in this study included a relatively higher number of patients who underwent RFA for pHPT than in other studies in the literature, which can be cited as its primary strength. However, this study also had some limitations. The first limitation was its retrospective design, whereas the second limitation was the absence of pathology results, which might have masked the presence of adenocarcinoma cases. The study’s controversial findings, which differed from the relevant findings reported in the literature, might be attributed to its retrospective design or technical insufficiency, which may have prevented complete adenoma destruction. Moreover, we did not collect the data regarding complications due to the retrospective nature of this study.

In conclusion, RFA may be considered an alternative method for treating solitary parathyroid adenoma-related pHPT. Nevertheless, prospective studies with larger sample sizes and more extended follow-up periods are needed to establish the feasibility and safety of RFA for the treatment of pHPT.

## Figures and Tables

**Table 1:** Distribution of the postoperative serum calcium and PTH levels.

Operation process		Serum calcium (mg/dL)	PTH (pg/mL)
**Baseline**†,‡		10.9 ± 0.7 10.9 [9.5–12.5]	208.4 ± 163.0 150.0 [74.0–1068.0]
**Postintervention**†,‡	Immediate	10.4 ± 0.8 10.4 [8.9–12.6]	74.9 ± 94.6 39.0 [2.0–450.0]
	1 week	9.9 ± 0.6 9.9 [9.1–12.2]	109.8 ± 68.4 108.2 [31.6–411.0]
	1 month	9.9 ± 0.7 9.9 [8.6–11.4]	134.5 ± 74.3 133.0 [29.0–382.0]
	3 months	9.7 [8.1–12.2] 9.7 [8.1–12.2]	108.2 [9.6–351.0] 108.2 [9.6–351.0]
	6 months	11.0 ± 5.4 10.7 [8.6–12.4]	117.6 ± 84.6 115.2 [15.0–597.0]
	1 year	9.8 ± 0.9 9.8 [8.0–12.8]	103.7 ± 53.1 103.6 [17.4–275.0]
**p-values**		**< 0.001**	**< 0.001**

† mean ± standard deviation,

‡ median [min–max]

PTH: parathyroid hormone

Friedman test

**Table 2:** Frequency of patients with normal serum calcium, PTH, and both serum calcium and PTH levels.

		Serum calcium < 10.5 mg/dL	PTH < 89 ng/mL	Serum calcium < 10.5 mg/dL and PTH < 89 ng/mL	Serum calcium < 10.5 mg/dL and PTH ≥ 89 ng/mL
**Baseline** §		11 (25.6)	3 (7.0)	3 (7.0)	11 (25.6)
**Postintervention** §	Immediate	24 (55.8)	32 (74.4)	20 (46.5)	4 (9.3)
	1 week	38 (88.4)	14 (32.6)	14 (32.6)	24 (55.8)
	1 month	34 (79.1)	9 (20.9)	9 (20.9)	25 (58.1)
	3 months	39 (90.7)	9 (20.9)	9 (20.9)	30 (69.8)
	6 months	21 (48.8)	9 (20.9)	9 (20.9)	12 (27.9)
	1 year	37 (86.0)	15 (34.9)	13 (30.2)	24 (55.8)

§ n (%)

PTH: parathyroid hormone

## Data Availability

Because of privacy regulations and participants’ informed consent, data cannot be made freely available in a public repository. Anonymized data will be shared by the corresponding author on reasonable request.

## References

[1] Li X Tufano RP Russell JO Yan L Xiao J Ultrasound-Guided Radiofrequency Ablation for the Treatment of Primary Hyperparathyroidism: An Efficacy and Safety Study Endocrine Practice 2021 27 12 1205 1211 10.1016/j.eprac.2021.07.012 34311118

[2] Wei Y Peng CZ Wang SR He JF Peng LL Microwave ablation versus radiofrequency ablation for primary hyperparathyroidism: a multicenter retrospective study Internal Journal of Hyperthermia 2021 38 1 1023 1030 10.1080/02656736.2021.1945689 34219596

[3] Ying W Zhen-Long Z Xiao-Jing C Li-Li P Yan L A study on the causes of operative failures after microwave ablation for primary hyperparathyroidism European Radiology 2021 31 6522 6530 10.1007/s00330-021-07761-9 33651201 PMC8379100

[4] Khandelwal AH Batra S Jajodia S Gupta S Khandelwal R Radiofrequency Ablation of Parathyroid Adenomas: Safety and Efficacy in a Study of 10 Patients Indian Journal of Endocrinology and Metabolism 2020 24 6 543 550 10.4103/ijem.IJEM_671_20 33643872 PMC7906106

[5] Hussain I Ahmad S Aljammal J Radiofrequency Ablation of Parathyroid Adenoma: A Novel Treatment Option for Primary Hyperparathyroidism AACE Clinical Case Reports 2021 7 3 195 199 10.1016/j.aace.2021.01.002 34095487 PMC8165122

[6] Sormaz IC Poyanlı A Açar S İşcan AY Ozgur İ The Results of Ultrasonography-Guided Percutaneous Radiofrequency Ablation in Hyperparathyroid Patients in Whom Surgery Is Not Feasible CardioVascular and Interventional Radiology 2017 40 4 596 602 10.1007/s00270-016-1544-6 28062897

[7] Chen Z Cheng L Zhang W He W Ultrasound-guided thermal ablation for hyperparathyroidism: current status and prospects Internal Journal of Hyperthermia 2022 39 1 466 474 10.1080/02656736.2022.2028907 35271788

[8] Wei Y Peng CZ Wang SR He JF Peng LL Effectiveness and Safety of Thermal Ablation in the Treatment of Primary Hyperparathyroidism: A Multicenter Study Journal of Clinical Endocrinology and Metabolism 2021 106 9 2707 2717 10.1210/clinem/dgab240 33846740 PMC8372654

[9] Ye J Huang W Huang G Qiu Y Peng W Efficacy and safety of US-guided thermal ablation for primary hyperparathyroidism: a systematic review and meta-analysis Internal Journal of Hyperthermia 2020 37 1 245 253 10.1080/02656736.2020.1734673 32138558

[10] Ha EJ Baek JH Baek SM Minimally Invasive Treatment for Benign Parathyroid Lesions: Treatment Efficacy and Safety Based on Nodule Characteristics Korean Journal of Radiology 2020 21 12 1383 1392 10.3348/kjr.2020.0037 32767864 PMC7689148

[11] Appelbaum L Goldberg SN Ierace T Mauri G Solbiati L US-guided laser treatment of parathyroid adenomas Internal Journal of Hyperthermia 2020 37 1 366 372 10.1080/02656736.2020.1750712 32308070

[12] Yazdani AA Khalili N Siavash M Shemian A Goharian AR Ultrasound-guided ethanol injection for the treatment of parathyroid adenoma: A prospective self-controlled study Journal of Research in Medical Sciences 2020 25 1 93 10.4103/jrms.JRMS_553_19 33273938 PMC7698383

[13] Shenoy MT Menon AS Nazar PK Moorthy S Kumar H Radiofrequency Ablation Followed by Percutaneous Ethanol Ablation Leading to Long-Term Remission of Hyperparathyroidism Journal of the Endocrine Society 2017 27 1 6 676 680 10.1210/js.2017-00094 29264521 PMC5686659

[14] Chai HH Zhao Y Zeng Z Ye RZ Hu QH Efficacy and Safety of Ultrasound-Guided Radiofrequency Ablation for Primary Hyperparathyroidism: A Prospective Study Korean Journal of Radiology 2022 23 5 555 565 10.3348/kjr.2021.0716 35506529 PMC9081691

[15] Erturk MS Cekic B Sarı IK Pamuk BO Microwave ablation as an efficient therapy for primary hyperparathyroidism: Efficacy and predictors of treatment success The International Journal of Clinical Practice 2021 75 10 e14580 10.1111/ijcp.14580 34185346

[16] Peng CZ Chai HH Zhang ZX Hu QH Zeng Z Radiofrequency ablation for primary hyperparathyroidism and risk factors for postablative eucalcemic parathyroid hormone elevation Internal Journal of Hyperthermia 2022 39 1 490 496 10.1080/02656736.2022.2047231 35285391

[17] Korkusuz H Wolf T Grünwald F Feasibility of bipolar radiofrequency ablation in patients with parathyroid adenoma: a first evaluation Internal Journal of Hyperthermia 2018 34 5 639 643 10.1080/02656736.2018.1453552 29607692

[18] Bilezikian JP Brandi ML Eastell R Silverberg SJ Udelsman R Guidelines for the management of asymptomatic primary hyperparathyroidism: summary statement from the Fourth International Workshop The Journal of Clinical Endocrinology & Metabolism 2014 99 10 3561 9 10.1210/jc.2014-1413 25162665 PMC5393490

[19] Wei Y Peng L Li Y Zhao ZL Yu MA Clinical Study on Safety and Efficacy of Microwave Ablation for Primary Hyperparathyroidism Korean Journal of Radiology 2020 21 5 572 581 10.3348/kjr.2019.0593 32323502 PMC7183824

[20] Zhang M Gao Y Zhang X Ding Z Wang X Evaluation of efficacy of ultrasound-guided microwave ablation in primary hyperparathyroidism Journal of Clinical Ultrasound 2022 50 2 227 235 10.1002/jcu.23134 34984687 PMC9303728

[21] Liu F Yu X Liu Z Qiao Z Dou J Comparison of ultrasound-guided percutaneous microwave ablation and parathyroidectomy for primary hyperparathyroidism Internal Journal of Hyperthermia 2019 36 1 834 839 10.1080/02656736.2019.1645365 31452422

[22] Ye J Huang W Huang G Qiu Y Peng W Efficacy and safety of US-guided thermal ablation for primary hyperparathyroidism: a systematic review and meta-analysis Internal Journal of Hyperthermia 2020 37 1 245 253 10.1080/02656736.2020.1734673 32138558

[23] Xu SY Wang Y Xie Q Wu HY Percutaneous sonography-guided radiofrequency ablation in the management of parathyroid adenoma Singapore Medical Journal 2013 54 7 e137 e140 23900476 10.11622/smedj.2013092

[24] Sormaz IC Poyanlı A Açar S İşcan AY Ozgur İ The Results of Ultrasonography-Guided Percutaneous Radiofrequency Ablation in Hyperparathyroid Patients in Whom Surgery Is Not Feasible CardioVascular and Interventional Radiology 2017 Apr 40 4 596 602 10.1007/s00270-016-1544-6 28062897

[25] Ni WJ Chu XQ Lu CY Chen GF Han X Effectiveness and safety of ultrasound-guided microwave ablation for the treatment of primary hyperparathyroidism in 12 patients with parathyroid adenoma Zhonghua Nei Ke Za Zhi 2021 60 10 904 907 Chinese. 34551480 10.3760/cma.j.cn112138-20201111-00935

